# A Short-Term Longitudinal Investigation of the Perceived Hookup Attitude of Gender-Specific Close Friends and Hookup Behavior

**DOI:** 10.3389/fpsyg.2020.01410

**Published:** 2020-07-14

**Authors:** Ke Yu, Yong Zheng

**Affiliations:** ^1^Center for Studies of Education and Psychology of Minorities in Southwest China, Southwest University, Chongqing, China; ^2^Sichuan University of Culture and Arts, Mianyang, China; ^3^Faculty of Psychology, Southwest University, Chongqing, China

**Keywords:** hookup, peer norms, hookup attitudes, casual sex, gender-specific

## Abstract

Research suggests that peer injunctive norms (i.e., perceived attitudes) have an indirect effect on youth’s behavior. Few studies have explored the underlying mechanism of the relationship between the perceived attitude of gender-specific close friends and hookup behavior. Following the social norm approach and the social learning theory, a person’s own hookup attitude and their emotional reaction after a hookup would influence this relationship as mediators. We therefore examined the association between perceived hookup attitudes of students’ same-sex/opposite-sex close friends and the number of hookup partners via mediated variables (i.e., own hookup attitude and affect following a recent hookup) in Chinese college students (*N* = 314) who completed self-report measures at 6-month intervals. The results of a path analysis showed that students’ own attitudes toward hookups and negative affect following a hookup significantly mediated the association between perceived hookup attitudes of same-sex close friends and the number of hookup partners over 6 months. These findings indicate that same-sex close friends’ injunctive norms were more strongly associated with future hookup behavior, as compared to opposite-sex friends’ injunctive norms. To better understand the relationship between peer norms and hookup behavior, both students’ hookup attitudes and negative emotional responses should be considered.

## Introduction

A hookup is a commonly used term to describe casual sexual behavior, ranging from kissing to intercourse, with a partner with whom there is no current romantic commitment and with whom no future romantic commitment is expected (Lewis et al., 2013). Hookups have become a part of campus culture in Western society. Western scholars have different views on hooking up. Some previous studies have demonstrated the potential benefits of hookups. There is emerging evidence that hooking up may have some benefits for adults in terms of satisfaction, such as increased self-esteem, sexual pleasure, and feeling attractive (Owen and Fincham, 2011; Lewis et al., 2012; Owen et al., 2014; Strokoff et al., 2015). Nonetheless, multiple studies indicate that hooking up involves risky, physically unhealthy behavior and outcomes, including negative emotional reactions (e.g., regret and embarrassment; Fisher et al., 2012; Lewis et al., 2012; Bachtel, 2013), unplanned pregnancy, sexually transmitted diseases (STDs) infection, and non-consensual sex (e.g., Downing-Matibag and Geisinger, 2009; Fielder and Carey, 2010; Garcia et al., 2012; Napper et al., 2015).

The young Chinese generation has been exposed to a series of sexually related social phenomena in China, such as the growth of single-gender families, the weakening of intergenerational transmission of sexual morality, and a decline in the rate of marriage (Cameron et al., 2013; Pan and Hou, 2013; Huang and Yi, 2015). In recent years, increasingly widespread Internet use, mass media, and consumer culture have conveyed messages that describe, praise, and guide various popular sexual subcultures. In this social context, hooking up may become fashionable among Chinese emerging adults (Huang et al., 2014; Pan and Hou, 2013; Jiang et al., 2018; Li et al., 2019). For example, one study of the younger Chinese generation found that having casual sex (e.g., sex with multiple partners) was significantly more common in 2010 compared to 2006 (Huang et al., 2014). The existing research on Chinese college students’ risky sexual behaviors has shown that the degree of approval for hooking up is increasing (Jiang et al., 2018). However, because of the lack of sex education in China, adolescents and young adults may not be provided with correct sexual knowledge and guidance and have limited understanding of the consequences of hooking up, which might lead to greater risks (e.g., sexual health problems). For example, the prevalence of STIs, including syphilis and chlamydia, and unwanted pregnancy continues to increase, all of which correlate with risky sexual behavior (e.g., sex with multiple partners) in China (Chen et al., 2011; Huang, 2016). Better understanding of the predictors and mediators of hookup behavior (e.g., hookups with multiple partners) may provide information for prevention and intervention efforts in hookups among Chinese college students.

Conventional college-aged students are in the stage of emerging adulthood, which is a key period of sexual exploration (Arnett, 2000). During this developmental stage, relationships with peers may be particularly vital, influential, and salient (Lefkowitz et al., 2004). Peer influence has a greater impact on personal behavior than other influences, such as cultural, personality, and religious factors (Berkowitz, 2004). The role of peer attitudes and behaviors in influencing Chinese college students’ sexual behavior (e.g., risky sexual behavior) may be crucial. This study focuses on the relationship between peer influence and hookup behavior among Chinese college students. Scholars point out that the attitudes and behaviors of a young person’s peers tend to be similar to their own attitudes and behaviors (e.g., Heilbron and Prinstein, 2008; Brechwald and Prinstein, 2011). This homophily might occur because youths tend to adopt similar attitudes and behaviors to their peers (i.e., socialization effects). Theories based on identity development, social learning, and social norms are concerned mainly with mutual socialization processes (i.e., peer influence) to explain peer homophily in youth behavior. Some scholars suggest that these peer influences are based less on their peers’ real beliefs and behaviors (i.e., “actual norms”) than on what they think others believe and do (i.e., “perceived norms”). Research has also indicated that, regardless of the actual norms, perceptions of peer norms may contribute significantly to individuals’ risky behaviors (e.g., alcohol abuse and drug use), as their behavior is consistent with what they believe to be their peers’ expectations (Perkins et al., 1999).

Existing literature has explored the influence of perceived norms toward hooking up on hookup behavior and consequences. Previous research on the association between gender-specific norms and risky behavior among college students has focused on alcohol use (e.g., Lewis and Neighbors, 2004). Gender-specific norms are comprised of opposite-sex norms (i.e., perceptions of typical behavior by opposite-sex peers) and same-sex norms (i.e., perceptions of this behavior by same-sex peers) (Lewis and Neighbors, 2004). Korcuska and Thombs (2003) suggested that alcohol use in both genders was best explained by same-sex peer drinking norms. Lewis et al. (2007) explored the gender-specific normative misperceptions of risky sexual behavior and discovered that college students perceived their opposite-sex peers to have fewer multiple sexual partners and a lower frequency of casual sexual intercourse than perceived by their same-sex peers. These conclusions are supported by both Social Impact Theory (Latane, 1981) and Social Comparison Theory (Festinger, 1954), which assert that more socially distal normative referents should have less impact on an individual’s behavior than more socially proximal normative referents. These theories also predict that the strength of the association between hookup injunctive norms (i.e., perceived hookup attitudes) and hooking up may be due to the social and intimate distance of peers (Barriger and Vélez-Blasini, 2013; Napper et al., 2014; Montes et al., 2017). For example, a study showed that injunctive norms were poor predictors of hookup behavior, perhaps because they are based on socially distal reference groups, such as a “typical student” (Barriger and Vélez-Blasini, 2013). This result is in accordance with other research (e.g., Montes et al., 2017; Neighbors et al., 2008). Specifically, the most influential hookup injunctive norms are those that characterize hookups by socially proximal referents (e.g., close friends) rather than more distal referents (e.g., typical students on campus).

However, previous studies have not evaluated how gender-specific injunctive norms (i.e., perceptions of same-sex/opposite-sex peer attitudes) influence hookup behavior (i.e., number of hookup partners). A meta-analysis by van de Bongardt et al. (2015) about the association between peer norms and adolescent sexual behavior showed that injunctive norms mainly have indirect effects on adolescent behavior. This result is consistent with previous longitudinal research (Fielder et al., 2013; Napper et al., 2014). Social learning theory (Bandura, 1971) suggests that learning can occur via observational learning, that is, imitation, or role modeling from valued social referents, such as close friends. According to this theory, the stronger a close peer’s approval of a certain behavior, the more the behavior is considered to be functional and correct, thereby influencing one’s own attitude or expectations toward the behavior (Cialdini and Trost, 1998; Fekadu and Kraft, 2002; Berkowitz, 2004). In addition, social learning theory points out that reinforcement is important for decisions about whether to perform a behavior. More specifically, an individual might avoid behaviors that lead to negative outcomes while being more likely to imitate and increasingly perform behaviors with more positive consequences. Owen et al. (2011) suggested that young adults with positive emotional responses after a prior hookup might have an increased likelihood of hooking up in the future, while negative responses after a prior hookup might decrease the likelihood of hooking up in the future. Furthermore, few studies have examined the underlying mechanism of the relationship using longitudinal data. Therefore, based on social learning theory and a social norm approach, we hypothesized that the association between the perceived hookup attitudes of college students’ same-sex/opposite-sex close friends and hookup partners over a 6-month period would have an indirect relationship, which was influenced by several intervening variables (e.g., own hookup attitude and emotional reaction after a recent hookup).

Previous studies have shown that the perceived hookup attitudes of proximal peers are more strongly associated with a person’s own attitudes toward hooking up than those of distal peers (e.g., Napper et al., 2014). Injunctive norms are frequently referred to as subjective norms, which are important aspects of the Theory of Planned Behavior (Ajzen, 1991) and the Theory of Reasoned Action (Fishbein and Ajzen, 1975). These theories suggest that the intention to engage in an action partly depends on the perceived approval of important others. Napper et al. (2014) pointed out that perceived approval of friends and parents predicted personal approval more strongly than the perceived approval of typical students. This result is consistent with research on broader injunctive norms (LaBrie et al., 2010; Neighbors et al., 2008). Specifically, the association between the perceived attitudes of others (i.e., close peers, school peers, and parents) was found to predict respondents’ own hookup attitudes, especially among proximal reference groups whose attitude may be more relevant and salient. We hypothesized that the perceived attitudes of same-sex/opposite-sex close friends can predict respondents’ own attitudes, and the perceived attitudes of close friends of the same sex would be more strongly predictive than those of the opposite sex.

College students’ hookup attitudes are related to emotional responses following a recent hookup. Some scholars have pointed out that emotional reactions mediate the relationship between hookup attitudes and hookup behaviors (Owen et al., 2010; Lewis et al., 2012). For example, negative emotional reactions were associated with less general approval of hooking up, which reduced the occurrence of this behavior. Approval of hookup behavior was positively related to positive emotional reactions and negatively related to negative emotional reactions (Lewis et al., 2012). Owen et al. (2010) indicated that an attitude that was less approving of hookups was associated with more negative emotional reactions. It may be that holding negative hookup attitudes and then engaging in hookup behavior despite this attitude may create an internal conflict that causes negative emotional responses. Therefore, we hypothesized that Chinese college students’ own hookup attitudes would be positively related to positive emotional reactions and negatively associated with negative emotional reactions after a recent hookup.

Furthermore, emotions play a vital role in human sexual strategies (Townsend and Wasserman, 2011). Emotional responses may affect one’s level of interest and engagement in future hooking up. Specifically, if an individual’s feelings and memories are highly negative following hooking up, they may discourage a person from engaging in future hookups; if a person often experiences positive affect following a hookup, this may motivate them to try to repeat the experience (Fielder and Carey, 2010). We hypothesized that positive reactions would be positively associated with hooking up with multiple partners within the 6 months of the study period; specifically, negative affect would be associated with a lower number of hookup partners within the 6 months in the current study.

The extant literature demonstrates that previous hookup behavior can predict future hookup behavior (Owen et al., 2011; Fielder et al., 2013; Katz and Schneider, 2013; Olmstead et al., 2013). Katz and Schneider (2013) suggested that previous hookup behavior is the best predictor, by far, of future hookups. Controlling for previous behavior is therefore important to identifying factors that predict hookups beyond this strong predictor (Fielder et al., 2013). Therefore, number of hookup partners reported at Time 1 was a vital control variable in the current study. In addition, several important demographic factors were sex (e.g., Manning et al., 2005; Owen et al., 2010), year in school (e.g., Bogle, 2008; Olmstead et al., 2013), and age (e.g., Napper et al., 2014; Montes et al., 2017), which were also controlled in the current study.

### The Current Study

**FIGURE 1:**
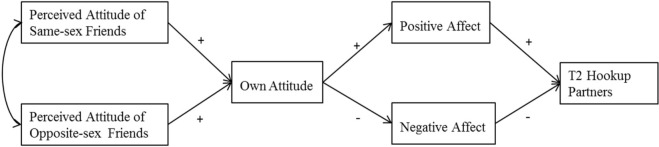
Conceptual model of current research hypotheses.

We employed a framework that combines antecedents with future hookup behaviors, which is described in the conceptual model (see Figure 1). More specifically, we proposed the following hypotheses. H1: we hypothesized that the pathway through which the perceived hookup attitudes of same-sex close friends and opposite-sex close friends would be associated with the number of hookup partners over a 6-month period would be via college students’ own attitudes toward hooking up and emotional reaction after a recent hookup. H2: the perceived attitudes of same-sex/opposite-sex close friends would be positively associated with respondents’ own hookup attitudes. H3: respondents’ own hookup attitudes would be positively related to positive emotional reactions following a recent hookup and negatively related to negative emotional reactions. H4: positive emotional reactions following a recent hookup would be positively associated with the number of hookup partners over a 6-month period, and negative affect would be negatively associated with the number of hookup partners over a 6-month period.

Few studies have used longitudinal studies to explore the relationship between, and the mechanism for, perceived hookup attitudes of gender-specific close friends and hookup behaviors (i.e., number of hookup partners). In addition, few scholars have paid attention to risky sexual behavior among Chinese college students. The current study aims to clarify how the perceived attitudes of socially proximal reference groups are related to future number of hookup partners, as well as the mechanisms associated with these relationships among Chinese college students. It is believed that the findings will provide more specific knowledge and opportunities for prevention of and intervention for risky sexual behavior, especially in China.

## Materials and Methods

### Participants and Procedures

A sample of Chinese college students in mainland China were invited to participate in a longitudinal study during October 2018 (T1) and May 2019 (T2). The questionnaire was administered via a professional survey website, *Wenjuanxing*^1^. Prior to answering questions regarding hooking up, hookups were broadly defined for participants as “Some people say that a hookup is a physical encounter between two people without necessarily expecting anything further (e.g., no plan or intention to do it again)” (Owen et al., 2011). Participants (*N* = 463, 49% female) who reported having experienced at least one hookup in the past year, and who completed an assessment of their attitudes toward hooking up, hookup injunctive norms, and emotional reaction following a recent hookup (T1), were deemed eligible for an additional survey 6 months after T1 data collection (T2). All initial respondents were contacted again for a T2 follow-up; 321 (48% female) college students completed T2 (69% retention rate).

After excluding data for students with incomplete responses, the final sample included 314 students (47.8% female) who completed both T1 and T2 measures, aged 18–25 years (*M* = 20.59, SD = 1.48), including Freshman (13.9%), Sophomore (31%), Junior (36.1%), and Senior (19%) years. In addition, compared to those who did not complete T2, there were no significant differences in injunctive hookup norms, own hookup attitude, emotional reaction after a hookup, or number of hookup partners in the past year.

### Measures

#### Hookup Injunctive Norms

At T1, participants were asked these questions: “How much do you think your same-sex closest friends approve of hooking up?” and “How much do you think your opposite-sex closest friends approve of hooking up?” Responses were measured on a 7-point Likert scale ranging from 1 (Strongly Disapprove) to 7 (Strongly Approve).

#### Own Hookup Attitude

At T1, participants were asked: “How much do you approve of hooking up?” Responses were measured on a 7-point Likert scale ranging from 1 (Strongly Disapprove) to 7 (Strongly Approve).

#### Positive and Negative Affect Related to Most Recent Hookup

The measure used to assess hookup affect resulting from the most recent hookup was based on previous research (i.e., Owen et al., 2010; Lewis et al., 2012). At T1, participants were asked, “Please indicate the extent to which you experienced the feeling or emotions below as a result of this hookup. This hookup made me feel…” followed by five positive (happy, desirable, attractive, carefree, and excited) and five negative items (regretful, ashamed, confused, upset, and depressed). Affect was measured on a 5-point Likert scale ranging from 1 (Not at all) to 5 (Very much). Higher scores represent either more positive or more negative affect. For positive and negative hookup-related affect, Cronbach’s alphas in this study were 0.90 and 0.83, respectively.

#### Hookup Behavior

At T1, participants were asked how many people they had hooked up with in the past year. At T2, participants were asked how many people they had hooked up with in the past 6 months. Response options ranged from 0 to 6 or more.

### Analytic Plan

Prior to analysis, SPSS was used to examine the variables’ distributional properties to assess the hypotheses related to path analysis (i.e., normality, no multicollinearity) (Tabachnick and Fidell, 2007). Skewness and kurtosis analyses indicated that all independent variables were normally distributed. Examining the variance inflation factor (VIF) for each independent variable assessed multicollinearity and singularity. Variance inflation factors for all variables were less than 2, confirming that multicollinearity was within an acceptable range.

Data analyses were conducted with Mplus 7.0 (Muthén and Muthén, 2012) and maximum likelihood was estimated. As model χ^2^ is impacted by the sample size and may lead to significance even when the model is minimally mis-specified (Marsh et al., 2004), Tucker–Lewis Index (TLI), the comparative fit index (CFI), standardized root mean square residual (SRMR), and root mean square error approximation (RMSEA) were also used to evaluate overall model-data fit. Values smaller than 0.06 and 0.08 for RMSEA and SRMR, and greater than 0.95 for CFI and TLI, suggest good model fit (Hu and Bentler, 1999). The indirect paths from hookup attitude of same-sex close friends and hookup attitude of opposite-sex close friends to hookup partners reported at T2 were examined with bootstrapping procedures (Preacher and Hayes, 2008). The bootstrapping analyses of the indirect pathways were conducted with 5,000 bootstraps and 95% confidence intervals (CI).

## Results

### Preliminary Analyses

Means and SDs are reported in Table 1, and correlations are shown in Table 2. The hypothesis that bivariate relationships between most variables in the path model would be statistically significant was supported, which provided preliminary evidence that the hypothesized path model fits the data.

**TABLE 1:** Sample characteristics (*N* = 314).

Variable	Median	*M*	SD	Range
PASF	4.000 (4.000)	3.755 (4.537)	1.695 (1.770)	1–7
PAOF	4.000 (3.000)	4.047 (3.079)	1.848 (1.749)	1–7
Own Attitude	4.000 (5.000)	4.247 (5.067)	1.642 (1.406)	1–7
Positive Affect	3.600 (4.000)	3.425 (3.796)	0.967 (0.870)	1–5
Negative Affect	2.200 (1.800)	2.265 (1.848)	0.955 (0.734)	1–5
T1 Hookup Partners	2.000 (2.000)	2.260 (2.805)	1.323 (1.661)	1–6
T2 Hookup Partners	1.000 (1.500)	1.513 (2.067)	1.958 (2.137)	0–6

*The descriptive data for women is displayed out of brackets and men are show in the brackets. PASF = perceived attitude of same-sex friends; PAOF = perceived attitude of opposite-sex friends.*

**TABLE 2:** Zero-order correlations by sex (*N* = 314).

Variables	1	2	3	4	5	6	7	8
1. PASF	−	0.589**	0.627**	0.353**	−0.262**	0.178*	0.174*	0.237*
2. PAOF	0.242**	−	0.503*	0.296**	−0.272**	0.201*	0.144	0.125
3. Own Attitude	0.291**	0.317***	−	0.568**	−0.408**	0.251**	0.242**	0.166*
4. Positive Affect	0.152	0.170*	0.454**	−	−0.481**	0.194**	0.193*	0.202*
5. Negative Affect	−0.195*	–0.110	–0.089	−0.234**	−	−0.301**	−0.283**	–0.127
6. T1 Hookup Partners	0.119	0.189*	0.268**	0.248**	−0.290**	−	0.445**	–0.038
7. T2 Hookup Partners	0.005	–0.041	0.131	0.118	−0.199**	0.444**	−	0.136
8. Age	–0.022	−0.159*	0.034	0.029	0.072	0.012	0.180*	−

Correlations of study variables for men are displayed on the bottom half and women are show on the top of the diagonal. PASF, perceived attitude of same-sex friends; PAOF, perceived attitude of opposite-sex friends. *p < 0.05; **p < 0.01; ***p < 0.001.

### The Path Model: Attitude of Close Friends Toward Hookups and Number of Hookups

Our initial model fit was good: RMSEA = 0.047 (CI: 000–0.092), CFI = 0.983, TLI = 0.966, SRMR = 0.031, and χ^2^ = 11.854 (*df* = 7, *p* > 0.05). The likelihood ratio test (Chou and Bentler, 1990) showed that the model might not be improved by incorporating additional paths. Therefore, the initial path model was accepted [perceived same- and opposite-sex friends’ hookup attitudes, own hookup attitudes, and positive and negative affect after a recent hookup were all measured at the same time-point, therefore we examined the alternative model (e.g., perceived same-sex/opposite-sex hookup attitudes of close friends → hookup affect → own hookup attitude → T2 hookup partners). The results did not suggest a good index fit of the alternative model (e.g., RMSEA = 0.200 CI: 0.162–0.240, CFI = 0.740, TLI = 0.394, SRMR = 0.085, χ^2^ = 81.184, *df* = 6, *p* < 0.001) was not good], and further analyses were performed with this model. Additionally, we controlled for important demographic information and variables, including the number of hookup partners at Time 1, sex (0 = Male, 1 = Female) [we found sex differences in own attitude (*b* = -0.0.701, *p* < 0.001) and negative emotional reactions (*b* = 0.248, *p* < 0.01)], year in college (1 = Freshmen to 4 = Senior), and age.

### Path Analysis Results

**FIGURE 2:**
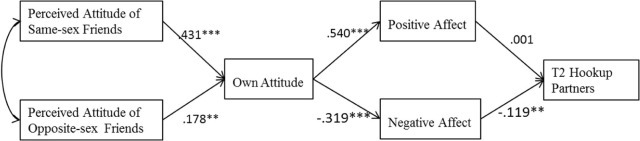
Path model with standardized path coefficients T1 hookup partners, sex, school year and age were controlled for in the analyses but are not included in the figure. **p < 0.01; ***p < 0.001.

This model and the associated findings are presented in Figure 2 and Table 3. The effect of perceived same-sex and opposite-sex close friends’ attitudes toward hooking up on participants’ reported number of hookup partners at T2 was hypothesized to operate indirectly via two mediator variables: individuals’ own hookup attitudes and affect following a recent hookup. Neither the direct effect of perceived hookup attitude of same-sex and of opposite-sex close friends on T2 hookup partners were significant (*b* = −0.028, *p* > 0.05; *b* = −0.085, *p* > 0.05) (see Table 3). In this model, both perceived same-sex close friends’ attitudes and perceived opposite-sex close friends’ attitudes were positively associated with college students’ own hookup attitudes. As hypothesized, the perceived attitudes of close friends of the same sex were a stronger predictor for own attitude, compared to those of the opposite sex. Moreover, participants’ own hookup attitudes were positively associated with positive affect following a recent hookup and negatively associated with negative affect. Only negative affect was found to be negatively associated with the number of hookup partners students reported at T2 (see Figure 2 and Table 3). Partially as hypothesized, significant indirect effects were shown regarding the effect of perceived hookup attitudes of only respondents’ same-sex close friends on the number of hookup partners via students’ hookup attitude and negative affect following a recent hookup (see Table 4). Alternatively, the indirect effect of perceived opposite-sex friends’ hookup attitudes on the number of partners at T2 was not significant.

**TABLE 3:** Determinants of hookup partners (*N* = 314).

Direct effects	*b*	SE	*P*
PASF→T2	–0.028	0.070	0.684
PAOF→T2	–0.085	0.066	0.196
Own Attitude→T2	0.111	0.089	0.210
Positive Affect→T2	–0.044	0.136	0.746
Negative Affect→T2	–0.292	0.135	0.031

*PASF, perceived attitude of same-sex friends; PAOF, perceived attitude of opposite-sex friends; T2, T2 hookup partners. Estimates are unstandardized path coefficients. All paths controlled for sex, school year, age, and T1 hookup partners.*

**TABLE 4:** Pathways from perceived hookup attitude of gender-specific close friends to hookup partners (*N* = 314).

	*b*	SE	CI 95%	*P*
**Indirect effects**				
1. PASF→Own Attitude→Negative Affect→T2	0.016	0.008	[0.001, 0.032]	0.037
2. PASF→Own Attitude→Positive Affect→T2	0.000	0.013	[-0.024, 0.025]	0.989
3. PAOF→Own Attitude→Negative Affect→T2	0.007	0.004	[0.000, 0.011]	0.063
4. PAOF→Own Attitude→Positive Affect→T2	0.000	0.003	[-0.010, 0.011]	0.989

*PASF, perceived attitude of same-sex friends; PAOF, perceived attitude of opposite-sex friends; T1, T1 hookup partners; T2, T2 hookup partners. Estimates are unstandardized path coefficients. All paths controlled for sex, school year, age, and T1 hookup partners.*

## Discussion

The present study tested pathways involving the perceived attitudes of participants’ gender-specific close friends and how they were related to the number of hookup partners over a 6-month period by examining a path model including two hypothesized intervening variables: respondents’ own hookup attitudes and emotional reaction after a recent hookup. The path model indicated that the perceived hookup attitudes of participants’ same-sex close friends were related to T2 number of hookup partners via participants’ own hookup attitudes and negative affect.

The perceived hookup attitudes of respondents’ same-sex/opposite-sex close friends could not directly predict college students’ hookup behavior over a 6-month period. This result is similar to the finding that injunctive norms have primarily indirect effects on youth behavior (e.g., Borsari and Carey, 2003; Baumgartner et al., 2011). Therefore, compared with other social norms, the social-psychological mechanism through which injunctive norms are related to young adults’ behavior may be a complicated and personalized process. The results of the current study clarify the social psychological process involved in gender-specific injunctive norms and demonstrated their exclusive connections with college students’ attitudes, emotional reactions, and behaviors regarding hooking up.

Both perceived hookup attitudes of college students’ same-sex and opposite-sex close friends were positively associated with their own attitude toward hooking up, and same-sex close friends seemed to have a more marked positive association. The intensity of influence was related to whether the gender of close friends was consistent with respondents’ own gender. Many developmental psychology studies have found that opposite-sex peers do not exert as strong an impact on adolescents’ developmental trajectories as same-sex peers (Liem and Martin, 2011). Moreover, individuals are more prone to interact, and develop friendships, with same-sex than opposite-sex peers (e.g., Maccoby and Jacklin, 1987; Bukowski et al., 1999; Feiring, 1999). Perceived hookup attitudes of students’ same-sex close friends were more strongly positively associated with their own hookup attitudes. Specifically, college students have more chances to talk about sexual topics (e.g., sexual experience) with their same-sex close friends. Same-sex close friends are likely to do so more easily and openly than opposite-sex close friends. For example, Lefkowitz et al. (2004) found that college women talk to their opposite-sex friends less comfortably and frequently about sex-related topics than with same-sex friends. Meanwhile, van de Bongardt et al. (2017) suggested that sexual communication with peers was related to perceived injunctive norms and could influence their own attitude toward hooking up. More specifically, college students interactively construct injunctive hookup norms through normative and deviant conversations with their closest friends (i.e., same-sex friends), which in turn have been shown to guide their own hookup attitudes.

Students’ own hookup attitudes reported at Time 1 were not significantly related to the number of hookup partners reported at Time 2. This result is consistent with existing research (e.g., Napper et al., 2014). Kirby (2002) found that having more permissive attitudes toward premarital sex was a risk factor for sexual initiation. However, in a longitudinal study of college students, attitudes about hooking up were a poor predictor of subsequent hookup behavior (Napper et al., 2014). Our results demonstrate that students’ own attitudes at Time 1 did not predict the number of hookup partners over a subsequent 6-month period. This indicates that the mechanism by which a person’s own hookup attitude influences their future hookup behavior may be more complex than previously suspected.

The current study examined the relationship between hookup attitude and emotional reactions. The results showed that more favorable attitudes toward hookups were related to less negative affect and greater positive affect. This aligns with previous research (e.g., Owen et al., 2010). Those with the most positive hookup attitudes and the strongest approval of sexual activity showed the greatest positive emotional response (Lewis et al., 2012). Moreover, this finding indicates that college students who feel within their “comfort zone” about hooking up are likely to have a more positive hookup response than those who feel outside their own comfort zone.

However, our results also indicate that, compared with positive emotional experience, negative experience acted as a more important predictor for hookup behavior. Negative emotional reactions following hooking up were negatively associated with multiple hookup partners at Time 2. There was no relationship between positive emotional response and hookup partners. Although this conclusion is inconsistent with previous research (e.g., Owen et al., 2011), it is understandable from the perspective of social learning theories. Negative emotional experience may have a greater impact on negative consequences, which can change the perception of hookups and prevent one from initiating hookup behavior. More specifically, negative hookup reactions tend to be associated with depressive symptoms and feelings of loneliness, which predicted higher levels of anxiety, while positive emotional reactions were not related to anxiety (Owen and Fincham, 2011; Winkeljohn Black et al., 2019). Previous studies found that college women who reported more negative hookup responses also showed lower levels of academic engagement (Owen et al., 2014), while positive hookup reactions did not predict academic engagement. Although hooking up is sometimes evaluated as a neutral or enjoyable experience, positive emotional responses may also be associated with negative consequences (Fielder and Carey, 2010; Owen and Fincham, 2011; Lewis et al., 2012). For example, Owen and Fincham (2011) discovered that, for college students who engaged in intercourse during a hookup, positive emotional responses were related to decreased condom use, which may lead to STDs or pregnancy. The mechanisms by which hookups may possibly influence youth well-being have not been clearly formulated, although several negative emotional reactions associated with hookups may offer potential explanations, such as more regret and less enjoyment than with romantic sex (Fielder and Carey, 2010) and failure to gratify the need for lasting and stable interpersonal connection (Baumeister and Leary, 1995). Therefore, it is not surprising that positive emotional experiences of a recent hookup were not related to the number of hookup partners, while negative emotional experiences predicted the number of hookup partners.

Previous research indicates that casual early sexual experiences exert an influence on later sexual and relational behaviors (Katz and Schneider, 2013). The current study shows that more negative emotional experiences following casual sexual encounters will lead to fewer hookup partners. These results suggest that focusing on negative emotional consequences following hookups may help us understand hookup behavior more clearly than focusing only on positive emotional experiences. This perspective requires further confirmation by future research. Moreover, future research can also explore the role of hookup consequences in the association between negative emotional experience and hookup behavior using longitudinal surveys.

The indirect effects of perceived attitude of opposite-sex close friends on hookup behavior on the number of partners at T2 was not significant. This conclusion is consistent with cross-cultural findings (e.g., Lewis et al., 2007). This further demonstrates that, for Chinese college students, perceptions of same-sex close friends’ hookup injunctive norms are more salient, as well as more precise, and are a better predictor of multiple hookup partners than perceptions of opposite-sex injunctive norms. This finding is supported by Social Comparison Theory and Social Impact Theory. More specifically, compared to more distal comparison referents (i.e., opposite-sex close friends), more socially proximal referents (i.e., same-sex close friends) would be considered more relevant and of greater influence. Similar patterns observed for alcohol use, normative perceptions for same-sex close friends may be particularly vital when assessing hookup behavior, as they represent more proximal referents than opposite-sex close friends. Perceived hookup attitudes would consequently precede, and therefore influence, the actual hookup behavior of college students.

### Limitations and Future Directions

This study has several limitations that can provide directions for future research. First, peer influence is referred to as a phenomenon characterized by the presence of both selection (choosing peers similar to oneself) and socialization (being influenced by one’s peers). Existing research has not fully explained the relationship between perceived gender-specific norms and number of hookup partners in terms of socialization theory. Future research could consider the role of selection in understanding the reciprocal, dynamic associations between socialization and selection in adolescent and adult peer relations (van de Bongardt et al., 2015). Second, the self-report measures employed here required students to reflect on a recent hookup experience, which may be difficult for some participants to recall. As we assessed participants’ emotional reaction after the most recent hookup, the time since the most recent hookup occurred would have varied from person to person. Thus, it is possible that there may have been some memory biases for individuals for whom the most recent hook up occurred more remotely in time than for others. Third, longitudinal studies could provide more explanation of the association between perceived hookup attitude of gender-specific close friends and number of hookup partners than cross-sectional research. However, the longitudinal period of this study was relatively short, and future research could attempt to be more sensitive to changes in the relationship between these variables to further elucidate the nature of this relationship. Fourth, in the current study, participants were provided with a standard definition that a hookup could range from kissing to intercourse. Future studies that examine the intimacy level of hookup behavior may provide more understanding of the relationships between close peer injunctive norms and hookup behavior. Barriger and Vélez-Blasini (2013) indicated that specific norms may be strong predictors of different types of intimacy during hooking up. In the future studies it would be useful to explore injunctive norms for type of intimacy during hookup (e.g., kissing, oral sex, and intercourse) separately. Fifth, existing research indicates that close friends’ injunctive norms can more strongly predict own hookup attitudes for men than for women (Napper et al., 2014). Furthermore, Lewis and Neighbors (2004) showed that perceived same-sex drinking norms are stronger predictors of alcohol consumption for women, as compared to men. Future research could examine the relationship between perceived gender-specific close friends’ norms and hooking up to explore the potential moderating effect of the respondents’ sex. Sixth, the current study did not focus on potential differences in hookup norm estimation (e.g., relationship status, race, and sex orientation). Future research should explore the possibility that individuals belonging to different groups (e.g., exclusive romantic relationship and non-exclusive romantic relationship) may systematically differ in their injunctive norms and number of hookup partners. Additionally, the study population was limited to a sample of Chinese college students only. Future research should attempt to include participants from other age groups (e.g., Chinese adolescents) or cultures.

## Conclusion

Our observations support the hypothetical path model we have developed, and our model makes a significant contribution to the existing literature and enriches the study on risky behaviors of Chinese college students. Our results indicate that future hookup behavior of college students was indirectly influenced by the perceived attitudes of same-sex close friends. Given the plausible differentiated effects of perceived same-sex and opposite-sex close friends injunctive norm on adults’ hookup behavior (Montes et al., 2017), differentiating opposite-sex from same-sex peer relationships lends greater clarity to the issue. Moreover, our examination of the hypothesized pathways suggests that negative affect plays a more vital role than positive affect. Understanding the pathways (i.e., psychosocial processes) through which emerging adults’ hookup behavior is associated with gender-specific peer norms is essential for prevention and intervention efforts targeted to promoting Chinese emerging adults’ healthy sexual behavior and well-being.

However, the state of sex education in China requires improvement. After 1985, the year in which the first case of HIV was confirmed in China, the Chinese government began to provide strong state support for sex education, which became part of the curriculum offered to adolescents and those nearing adulthood in school (Wang and Davidson, 2006). Although the government advocated sex education, no specific measures were provided regarding how to implement sex education programs. Pan and Huang (2011) randomly surveyed the total population of adolescents aged 14–17 years in China in 2010 and demonstrated that most adolescents reported that they had received little sexual knowledge from schools and families, apart from physiological knowledge (e.g., spermatorrhea, menstruation). The current research indicates that risky behavior in Chinese youth is cross-culturally consistent with the conclusions of Western studies. Chinese educators and scholars can learn from the implementation strategies of sex education in Western countries. For example, interventions that incorporate personalized feedback utilize peer norm correction strategies (Lewis et al., 2007). Providing personalized feedback to adolescents and young adults demonstrates that risky sexual behavior is not as approved of or as popular as people believe, which may lead students to reevaluate their own attitudes and behavior. These strategies are more likely to reduce risky behavior than to increase resistance or complacency in individuals engaged in these highly risky sexual behaviors.

## Data Availability Statement

The datasets generated for this study are available on request to the corresponding author.

## Ethics Statement

The studies involving human participants were reviewed and approved by the Ethics Committee of Southwest University. The patients/participants provided their written informed consent to participate in this study.

## Author Contributions

KY conducted the study, performed the data analyses, and wrote the manuscript. YZ advised on the execution and analysis of the research and cooperated in the editing of the manuscript. Both authors have contributed equally to the design of the research.

## Conflict of Interest

The authors declare that the research was conducted in the absence of any commercial or financial relationships that could be construed as a potential conflict of interest.

## Footnotes

1 www.wjx.cn/
